# Increasing incidence and mortality of infective endocarditis: a population-based study through a record-linkage system

**DOI:** 10.1186/1471-2334-11-48

**Published:** 2011-02-23

**Authors:** Ugo Fedeli, Elena Schievano, Dora Buonfrate, Giampietro Pellizzer, Paolo Spolaore

**Affiliations:** 1 SER - Epidemiological Department, Veneto Region, Italy; 2 Infectious Diseases Unit, Vicenza Hospital, Italy

## Abstract

**Background:**

Few population-based studies provide epidemiological data on infective endocarditis (IE). Aim of the study is to analyze incidence and outcomes of IE in the Veneto Region (North-Eastern Italy).

**Methods:**

Residents with a first hospitalization for IE in 2000-2008 were extracted from discharge data and linked to mortality records to estimate 365-days survival. Etiology was retrieved in subsets of this cohort by discharge codes and by linkage to a microbiological database. Risk factors for mortality were assessed through logistic regression.

**Results:**

1,863 subjects were hospitalized for IE, with a corresponding crude rate of 4.4 per 100,000 person-years, increasing from 4.1 in 2000-2002 to 4.9 in 2006-2008 (p = 0.003). Median age was 68 years; 39% of subjects were hospitalized in the three preceding months. 23% of patients underwent a cardiac valve procedure in the index admission or in the following year. Inhospital mortality was 14% (19% including hospital transfers); 90-days and 365-days mortality rose through the study years. Mortality increased with age and the Charlson comorbidity index, in subjects with previous hospitalizations for heart failure, and (in the subcohort with microbiological data) in IE due to Staphylococci (40% of IE).

**Conclusions:**

The study demonstrates an increasing incidence and mortality for IE over the last decade. Analyses of electronic archives provide a region-wide picture of IE, overcoming referral biases affecting single clinic or multicentric studies, and therefore represent a first fundamental step to detect critical issues related to IE.

## Background

Over the last years epidemiological characteristics of infective endocarditis (IE) have been changing in industrialized countries as a result of advances in medical practice: decreasing prevalence of rheumatic heart disease as a predisposing condition, increased longevity, increasing number of patients who undergo invasive procedures [1-5]. Therefore, the emerging population at risk for IE consists of patients with health care-associated infections (acquired during hospitalization or following invasive procedures performed in other health-care settings) [6-8], elderly patients with valvular sclerosis, patients with valvular prostheses, and haemodialysis patients [1,5,9]. Most studies have recently shown a trend towards increasing incidence of *Staphylococcus aureus *endocarditis [1,10,11].

Morbidity and mortality are still considerable [2,5,10]: the incidence of IE ranges within 3-10 episodes/100,000 person-year [2]; the in-hospital mortality rate of patients with IE varies from 9,6 to 26% [2]. Larger studies are usually multicentric surveys [1] with selected patients (e.g., only centers with cardiac surgery, with a high proportion of transferred patients) and limited follow-up (only in-hospital or short-term). On the other hand, few high quality population-based studies reviewed in 2007 [9] were heterogeneous as regards population size and demographic structure (male/female ratio ranging from 1.2/1 to 2.5/1, mean or median age ranging from 51 to 69 years), case and outcome definition (from inhospital mortality to 1 year mortality, with overall mortality ranging from 14% to 46% according to different definitions); crude incidence varied from 1.4 to 6.2 per 100,000.

The objective of our study was to provide population-based descriptive epidemiological data of IE in the Veneto Region (North-Eastern Italy) through linkage of electronic archives of hospital discharge records (HDR) and mortality records.

## Methods

The total population of the Veneto Region was 4,885,548 on January 1, 2009. In the Region there are about 65 hospitals (including public and private institutes) with an overall number of about 16,000 hospital beds for acute care; there are approximately 900,000 discharges from Veneto hospitals each year. One primary and up to five secondary discharge diagnoses, plus one primary and up to five secondary procedures are registered in the regional archive of HDRs, which includes all discharges from Veneto hospitals and all discharges of Veneto residents hospitalized outside the region. HDRs with a primary or secondary International Classification of Diseases, 9th Revision, Clinical Modification -versions 1997 and 2002- diagnosis code of IE (421.x = acute and sub-acute endocarditis; 98.84 = gonococcal endocarditis; 112.81 = candidal endocarditis) were extracted starting from 1 January 1999.

In order to identify a cohort of incident cases with adequate follow-up and to prevent double counting of the included subjects, the first hospitalization (day-case excluded) for IE in the years 2000-2008 was selected; patients already discharged in 1999 with a diagnosis of IE were removed (prevalent cases). Demographic (age, gender) and clinical information (Charlson comorbidity computed from discharge diagnoses [12]) were extracted from the selected HDR. For each subject, all hospitalizations in the year before the first admission for IE were traced and diagnostic and intervention codes analyzed in order to retrieve information on comorbidities and risk factors (cancer, diabetes mellitus, chronic renal failure, congestive heart failure, previous cardiac valve surgery). Furthermore, all hospitalizations in the year following the first admission for IE were selected in order to gain information on hospital transfers (zero or one day difference between discharge and subsequent admission for IE), overall in-hospital mortality, re-admissions, and cardiac valve surgeries. Finally, the cohort of hospitalized IE was linked to the regional mortality registry of the years 2000-2008 to estimate survival of patients; data were censored after a follow-up of 365 days.

The microbiologic aetiology of IE is rarely reported in HDR by means of diagnostic codes 38.x or 41.x. Additionally, six regional hospitals sent microbiological data to a common database in the years 2004-2006; information on isolates from blood cultures was extracted and linked to HDR to identify the causative microorganisms and their influence on outcomes in such small subset of the cohort.

The study was carried out on data routinely collected by health services and linkage was performed on anonymized records without any possibility of identification of individuals. The study was approved by the Institutional Review Board of the Vicenza Hospital.

Continuous variables are presented as medians with interquantile ranges; categorical variables are presented as frequencies/percentages. The presence of time trends across the time periods was assessed by means of the Chi-square test for linear trend or a non parametric trend test derived from the Wilcoxon rank-sum test, as appropriate. The influence of predictive variables on short term mortality (dependent variable = vital status at 90 days) was evaluated by computing the Odds Ratio (OR) with 95% Confidence Intervals (CI). Variables resulting statistically significant at univariate analysis were selected by two models of stepwise logistic regression, including or excluding the Charlson index (which already takes into account diseases reported in the index admission). Statistical analyses were performed using commercially available software (Stata version 9.1; SAS version 9.1).

## Results

After excluding prevalent cases, 1,863 residents in the Veneto Region were hospitalized for IE in the period 2000-2008. The number of incident IE increased from 562 in 2000-2002 to 700 in 2006-2008 (+25%), with a corresponding crude rate rising from 4.1 to 4.9 per 100,000 person-years (+17%; p = 0.003). Table 1 shows the distribution of demographic characteristics, risk factors and comorbidities in the selected population, and significant changes through 2000-2008. The male to female ratio was 1.7:1, and 60% of subjects were aged 65 and older with a significant increase across the study period. The median age (interquantile range) was 68 years (57-77) in the whole cohort, increasing from 66 years (54-74) in 2000-2002 to 70 years (58-78) in 2006-2008 (p < 0.001). Globally, the rate of IE in people aged 65 and older was 20.3 per 100,000 person-years in male and 10.9 per 100,000 in female subjects. About two out five subjects were hospitalized in the three months preceding the index admission; in 12% a diagnostic code for congestive heart failure was reported in the year before the onset of IE. Information retrieved both from the index and from prior hospitalizations showed that a relevant proportion of the cohort was affected by diabetes mellitus (16%), cancer (10%), and chronic renal failure (8%).

**Table 1:** Demographic and clinical characteristics of hospitalized subjects with infective endocarditis in 2000-2002, 2003-2005, 2006-2008, and p value of the Chi square test for linear trend across the study periods

	2000-2002 (n = 562)	2003-2005 (n = 601)	2006-2008 (n = 700)	p for trend
Age ≥ 65 years	52.31%	63.56%	63.43%	** < 0.001**
Females	39.15%	41.60%	33.00%	**0.017**
Chronic renal failure	7.30%	7.99%	8.86%	0.309
Cancer	8.90%	11.15%	10.00%	0.563
Diabetes mellitus	16.01%	17.80%	15.29%	0.673
Previous congestive heart failure	12.99%	12.31%	11.43%	0.396
Valve procedures, previous 12 months	6.76%	5.49%	6.29%	0.767
All hospitalizations, previous 3 months	39.15%	41.60%	35.57%	0.159
Charlson index > 0	47.51%	48.92%	47.86%	0.927

Table 2 shows the sparse microbiological data retrieved from diagnostic codes of HDR in the whole cohort (available in 502 subjects), and from blood isolates in the few hospitals participating to the microbiological archive (available in 106 subjects). Although raw (ICD9-CM codes can be hardly translated into a microbiological classification), limited, and related to different subsets of the cohort, the two sources of information are consistent in indicating that about 40% of IE were due to Staphylococci (mainly *S. aureus*), followed by Streptococci, Enterococci and Gram negatives.

**Table 2:** Microbiological data retrieved from secondary diagnostic codes in discharge records (n = 502), and from blood culture samples (six hospitals, 2004-2006: n = 106)

Hospital discharge records		Isolates from blood	
Microorganism (ICD9-CM)	n (%)	Microorganism	n (%)
*Staphylococci*	*210 (42%)*	*Staphylococci*	*41 (39%)*
*S. aureus *(38.11, 41.11)	87	*S. aureus*	31
Coagulase-negative Staphylococci (38.19, 41.19)	30	Coagulase-negative Staphylococci	7
*Staphylococcus spp *(38.1, 38.10, 41.1, 41.10)	93	*Staphylococcus spp*	3
*Streptococci + Enterococci*	*213 (42%)*	*Streptococci+Enterococci*	*43 (41%)*
*Streptococci*, all (38.0, 41.0x not 41.04)	186	*S. bovis*	11
		*S. mitis*	8
		Other *Streptococci*	11
*Enterococci*, all (41.04)	27	*Enterococcus faecalis*	10
		*Enterococcus spp*.	3
Gram-negative bacteria (38.4x, 41.3-41.7)	50 (10%)	Gram-negative bacteria	13 (12%)
Others (38.2, 38.3, 41.2, 41.8, 98.84, 112.81	29 (6%)	Others	9 (8%)

List of ICD-9-CM codes adopted:

38.1 Staphylococcal septicemia; 38.10 Staphylococcal septicemia, unspecified; 38.11 Staphylococcus aureus septicaemia; 38.19 Other staphylococcal septicemia

Bacterial infection in conditions classified elsewhere and of unspecified site: 41.1 Staphylococcus; 41.10 Staphylococcus, unspecified; 41.11 Staphylococcus aureus; 41.19 Other Staphylococcus

38.0 Streptococcal septicemia

Bacterial infection in conditions classified elsewhere and of unspecified site: 41.0 Streptococcus; 41.04 Group D [Enterococcus]

38.4 Septicemia due to other gram-negative organisms

Bacterial infection in conditions classified elsewhere and of unspecified site: 41.3 K. pneumoniae; 41.4 E. coli; 41.5 H. influenzae; 41.6 P. mirabilis; 41.7 Pseudomonas

38.2 Pneumococcal septicaemia; 38.3 Septicemia due to anaerobes

Bacterial infection in conditions classified elsewhere and of unspecified site: 41.2 Pneumococcus; 41.8 Other specified bacterial infections

98.84 gonococcal endocarditis; 112.81 candidal endocarditis

**Figure 1:**
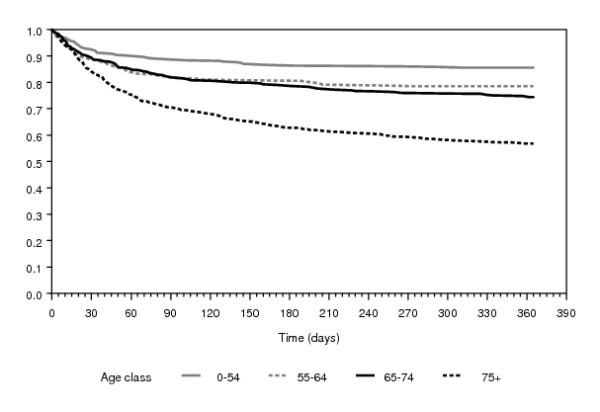
**Survival curves of subjects with infective endocarditis according to age class**.

Table 3 shows surgical treatment and outcomes of IE. The median length of stay increased from 23 to 33 days when including hospital transfers, with a rising trend through the study period. If the analysis was restricted to survivors, the median stay was 24 and 35 days excluding or including hospital transfers, respectively. Furthermore, a substantial proportion of subjects surviving the first hospitalization were re-admitted (transfers excluded) with a diagnostic code of IE within 1 year. 23% of subjects underwent a cardiac valve procedure in the index admission or in the following year; such percentage heavily decreased with age (< 55 yrs = 36%; 55-64 yrs = 32%, 65-74 yrs = 23%; ≥75 yrs = 10%; p for trend <0.001). Among subjects submitted to cardiac valve procedures, 37% were treated in the index admission, 24% following an hospital transfer, and 38% in a subsequent readmission within 1 year. The inhospital mortality was 14.3% when excluding hospital transfers, with only a slight and non significant increase through study years; it rose to 18.5% when transfers were accounted for. The linkage with mortality data showed that a substantial proportion of deaths happened between 30 and 90 days from the admission for IE (Figure 1); an increasing time trend in overall mortality was observed at 90 and 365 days of follow-up (Table 3).

**Table 3:** Hospitalization data and follow-up of subjects with infective endocarditis (IE) in 2000-2002, 2003-2005, 2006-2008, and p value of the test for linear trend across the study periods

	2000-2002 (n = 562)	2003-2005 (n = 601)	2006-2008 (n = 700)	p for trend
Median length of stay, including (excluding) hospital transfers	30 (23)	34 (23)	35 (25)	**0.024** (0.072)
In-hospital mortality, including (excluding) hospital transfers	16.9% (13.5)	19.3% (15.3)	19.1% (14.0)	0.328 (0.853)
Valve surgery in the index admission or within 1 year	23.5%	23.8%	22.0%	0.512
Repeated admissions with IE within 1 year among survivors	25.1%	25.2%	24.3%	0.645
90 days mortality	16.2%	20.8%	22.9%	**0.004**
365 days mortality	24.6%	28.8%	31.5%*	**0.013**

*data limited to 2006-2007

Figure 1 shows survival of subjects according to age: the survival curves of younger patients flatten after 60-90 days, while curves of older patients (especially those aged over 74) continue to drop until the end of follow-up probably due to several comorbidities.

The raise of incident cases observed among residents in the Veneto Region and the increase in 90-day fatality produced a growing mortality rate associated to IE, from 0.7 to 1.1 per 100,000 person-years (+65%; p < 0.001).

Table 4 shows the association of demographic and clinical variables already reported in Table 1 with 90 days mortality; the risk of death increased with age and the Charlson comorbidity index, in subjects hospitalized in the previous three months (only at univariate analysis), in those with a previous diagnosis of heart failure, and in patients with a mention of cancer and chronic renal failure. In the subsets of the cohort with information on microbiology, infections due to Staphylococci (n = 210/502 according to HDR) or to *S. aureus *(n = 31/106 according to blood isolates) were significantly associated to mortality, with an estimated OR (95% CI) equal to 3.0 (1.8-5.0) and to 4.2 (1.5-11.4), respectively.

**Table 4:** Variables associated to 90-days mortality: odds ratio (OR) with 95% Confidence Interval (CI), at univariate analysis, and at stepwise logistic regression including (Model 1) or excluding (Model2) the Charlson index.

	Univariate	Model 1	Model 2
	**OR (CI)**	**OR (CI)**	**OR (CI)**
	
Age (years)	1.03 (1.02-1.04)	1.03 (1.02-1.04)	1.03 (1.02-1.04)
Gender (females vs males)	0.89 (0.70-1.13)		
Chronic renal failure	1.83 (1.24-2.68)		1.50 (1.02-2.20)
Cancer	1.76 (1.23-2.49)		1.69 (1.20-2.38)
Diabetes mellitus	1.12 (0.81-1.52)		
Previous heart failure	2.24 (1.63-3.06)	1.85 (1.35-2.56)	1.80 (1.32-2.48)
Previous valve procedures	1.11 (0.67-1.77)		
Previous hospitalizations (3 mth)	1.35 (1.07-1.71)		
Charlson index 1 vs 0	2.19 (1.66-2.87)	2.00 (1.52-2.65)	
Charlson index ≥2 vs 0	2.78 (2.09-3.70)	2.44 (1.82-3.26)	

## Discussion

Our study demonstrates an increasing trend in incidence and mortality for IE in the Veneto Region over the last decade. The main limit of the study is the lack of validation of IE tracked by ICD9-CM discharge codes: such disease misclassification could have led to the inclusion of false positive cases and the exclusion of false negatives. Discharge diagnoses were chosen based on a pilot investigation carried out in recent years in a single Veneto hospital by chart review of discharges with a broader extraction of diagnostic codes, including as well as 421.x, 98.84, 112.81, also 93.2 (syphilitic endocarditis), 391.1 (acute rheumatic endocarditis), 424.9x (endocarditis, valve unspecified), 966.61 (infection and inflammatory reaction due to cardiac device implant and graft). IE diagnoses were validated according to the modified Duke criteria [13], demonstrating a positive predictive value and a sensitivity for IE of the selection applied in the present study equal to 91/123 = 81% and 91/98 = 93%, respectively (Pellizzer, personnel communication). To what extent such findings are applicable to the entire region and study period remains uncertain, but disease misclassification does not probably account for such sharp time trends found in our study; moreover, the incidence rate (4.4 per 100000 person-years) is in the range reported in literature and in particular it's similar to incidences estimated by multicenter prospective surveys conducted in other regions of Northern Italy [14,15]. Furthermore, since the period analyzed to exclude prevalent cases increases over time (minimum = 365 days in 2000), possibly not all prevalent cases have been deleted from early study years, leading to a small underestimation of the real increase of IE incidence; this effect was tested using a constant wash-out time of 365 days through the study period.

Our investigation is a large population-based survey spanning over several years that through record-linkage allows for a complete follow-up of IE cases as regards multiple outcomes: surgical interventions, hospital re-admissions, short-term and middle-term mortality. Descriptive data on incidence, surgical treatment, case-fatality obtained by the record-linkage system are within the range reported in the international literature. It must be remarked that in our analyses day 0 is the date of admission; as a consequence, health care-associated cases would have altered figures on length of stay and survival. However survival curves (except for the oldest age group) flatten after the first 60-90 days from admission; therefore 90-day mortality seems to be a good estimate of short term mortality associated to IE. Although recent studies report similar mortality rates in patients diagnosed and treated in tertiary care hospitals and those referred to tertiary centres from other hospitals [16,17], our data show that some usual outcome measures, such as simple in hospital mortality, could be inadequate since they miss a relevant burden of deaths occurring after discharge at home or in subsequent hospitalizations.

On the other hand, constraints in clinical data (risk factors, comorbidities, diagnostic work-out and medical therapy) and the almost complete lack of microbiological data derivable from HDR limit our interpretation. Although more accurate diagnostic techniques (such as a higher use of transesophageal echocardiography) can play a role in rising incidence rates [18,19], we cannot exclude that population at risk itself is increasing, considering the increasing share of elderly subjects among patients with IE.

According to literature, we confirm a high mortality associated to IE, particularly in older ages and in subjects with comorbidities [2,10,20-22]; a novel finding is the poor prognosis in subjects with prior hospitalizations for heart failure. However, the increasing trend in mortality can only partially be explained by the growing number of affected elderly patients. It must be enlighten that some well identified predictors of poor prognosis such as some echocardiographic findings (size of vegetations, presence of periannular complications, severity of valve dysfunction at diagnosis) and the presence of foreign material [2,18,23-25] couldn't be deduced from HDR. Moreover, we couldn't get differences in treatment protocols over time and among different hospitals.

In particular, our survey lacks information on indications for surgery; we found that 23% of patients underwent surgical treatment, with a probability sharply decreasing with age. Recent multicentric studies reported surgery in about half of cases [1,26]. However, some studies included only centres with cardiac surgery units, and the percentage of surgical therapy decreased when restricting analysis to patients admitted directly to study sites [1]; moreover they dealt with a younger population (median age under 60). Among population-based studies, the share of patients submitted to surgery ranged from 12.8% to 40% [9,14,15], with the exception of a survey in France where 49% of subjects were surgically treated [27]. Although there are still some areas of debate and individualized factors must be considered, it's been advocated that early surgery could be worth even in patients with high operative risk [27]; in general, in all IE cases, early surgical consultation is recommended [2,26].

The high proportion of patients with previous hospitalization may explain the consistent - although limited - microbiological data, being *S. aureus *the leading microorganism isolated. We also found that infections caused by Staphylococci are significantly associated to mortality, a finding consistent with literature [1,3,4,11,14,28]. Whether an increasing number and severity of comorbidities in subjects developing IE or, as previously hypothized [1,4,11], an increasing share of IE due to Staphylococci, could have led to the observed raise in mortality remains uncertain and deserves further investigations on a clinical basis.

## Conclusions

The study demonstrates an increasing incidence and mortality for IE over the last decade. Some usual outcome measures (in hospital mortality) miss a relevant burden of deaths occurring after discharge. Analyses through electronic archives allow to draw a region-wide picture of IE, overcoming those referral biases that unavoidably affect single clinic or multicentric studies, and therefore represent a first fundamental step to detect critical issues related to infective endocarditis.

## Competing interests

The authors declare that they have no competing interests.

## Authors' contributions

GP and SP designed the study and revised the manuscript, ES and UF collected data and performed statistical analyses, UF and DB wrote the first draft of the manuscript. All authors read and approved the final manuscript.

## Pre-publication history

The pre-publication history for this paper can be accessed here:

http://www.biomedcentral.com/1471-2334/11/48/prepub

## References

[B1] Murdoch DR Corey GR Hoen B Miró JM Fowler VG Jr Bayer AS Karchmer AW Olaison L Pappas PA Moreillon P Chambers ST Chu VH Falcó V Holland DJ Jones P Klein JL Raymond NJ Read KM Tripodi MF Utili R Wang A Woods CW Cabell CH International Collaboration on Endocarditis-Prospective Cohort Study (ICE-PCS) Investigators Clinical presentation, etiology, and outcome of infective endocarditis in the 21st century: the International Collaboration on Endocarditis-Prospective Cohort Study Arch Intern Med 2009 169 463 473 10.1001/archinternmed.2008.603 19273776 PMC3625651

[B2] The task force on the prevention, diagnosis, and treatment of infective endocarditis of the European Society of Cardiology (ESC) Guidelines on the prevention, diagnosis, and treatment of infective endocarditis (new version 2009) European Heart Journal 2009 30 2369 2413 10.1093/eurheartj/ehp285 19713420

[B3] Hill EE Herijgers P Herregods MC Peetermans WE Evolving trends in infective endocarditis Clin Microbiol Infect 2006 12 5 12 10.1111/j.1469-0691.2005.01289.x 16460540

[B4] Cabell CH Jollis JG Peterson GE Corey GR Anderson DJ Sexton DJ Woods CW Reller LB Ryan T Fowler VG Jr Changing patient characteristics and the effect on mortality in endocarditis Arch Intern Med 2002 162 90 94 10.1001/archinte.162.1.90 11784225

[B5] Moreillon P Que YA Infective endocarditis Lancet 2004 363 139 149 10.1016/S0140-6736(03)15266-X 14726169

[B6] Fernández-Hidalgo N Almirante B Tornos P Pigrau C Sambola A Igual A Pahissa A Contemporary epidemiology and prognosis of health care-associated infective endocarditis Clin Infect Dis 2008 47 1287 1297 18834314 10.1086/592576

[B7] Benito N Miró JM de Lazzari E Cabell CH del Río A Altclas J Commerford P Delahaye F Dragulescu S Giamarellou H Habib G Kamarulzaman A Kumar AS Nacinovich FM Suter F Tribouilloy C Venugopal K Moreno A Fowler VG Jr ICE-PCS (International Collaboration on Endocarditis Prospective Cohort Study) Investigators Health care-associated native valve endocarditis: importance of non-nosocomial acquisition Ann Intern Med 2009 150 586 594 19414837 10.7326/0003-4819-150-9-200905050-00004 PMC3625649

[B8] Lomas JM Martínez-Marcos FJ Plata A Ivanova R Gálvez J Ruiz J Reguera JM Noureddine M de la Torre J de Alarcón A Grupo Andaluz parael Estudio de las Infecciones Cardiovasculares (Andalusian Group for the Study of Cardiovascular Infections) at the Sociedad Andaluza de Enfermedades Infecciosas (SAEI) Healthcare-associated infective endocarditis: an undesirable effect of healthcare universalization Clin Microbiol Infect 2010 16 1683 1690 10.1111/j.1469-0691.2010.03043.x 19732086

[B9] Tleyjeh IM Abdel-Latif A Rahbi H Scott CG Bailey KR Steckelberg JM Wilson WR Baddour LM A systematic review of population-based studies of infective endocarditis Chest 2007 132 1025 1035 10.1378/chest.06-2048 17873196

[B10] Baddour LM Wilson WR Bayer AS Fowler VG Jr Bolger AF Levison ME Ferrieri P Gerber MA Tani LY Gewitz MH Tong DC Steckelberg JM Baltimore RS Shulman ST Burns JC Falace DA Newburger JW Pallasch TJ Takahashi M Taubert KA Committee on Rheumatic Fever, Endocarditis, and Kawasaki Disease; Council on Cardiovascular Disease in the Young; Councils on Clinical Cardiology, Stroke, and Cardiovascular Surgery and Anesthesia; American Heart Association; Infectious Diseases Society of America AHA Scientific Statement. Infective endocarditis. Diagnosis, antimicrobial therapy, and management of complications. A statement for healthcare professionals from the Committee on rheumatic fever, endocarditis, and Kawasaki disease, Council on cardiovascular disease in the young, and the Councils on clinical cardiology, stroke, and cardiovascular surgery and anesthesia, American Heart Association Circulation 2005 111 e394 e433 10.1161/CIRCULATIONAHA.105.165564 15956145

[B11] Fowler VG Jr Miro JM Hoen B Cabell CH Abrutyn E Rubinstein E Corey GR Spelman D Bradley SF Barsic B Pappas PA Anstrom KJ Wray D Fortes CQ Anguera I Athan E Jones P van der Meer JT Elliott TS Levine DP Bayer AS ICE Investigators Staphylococcus aureus endocarditis: a consequence of medical progress JAMA 2005 293 3012 3021 10.1001/jama.293.24.3012 15972563

[B12] Quan H Sundararajan V Halfon P Fong A Burnand B Luthi JC Saunders LD Beck CA Feasby TE Ghali WA Coding algorithms for defining comorbidities in ICD-9-CM and ICD-10 administrative data Med Care 2005 43 1130 1139 10.1097/01.mlr.0000182534.19832.83 16224307

[B13] Li JS Sexton DJ Mick N Nettles R Fowler VG Jr Ryan T Bashore T Corey GR Proposed modifications to the Duke criteria for the diagnosis of infective endocarditis Clin Infect Dis 2000 30 633 638 10.1086/313753 10770721

[B14] Cecchi E Forno D Imazio M Migliardi A Gnavi R Dal Conte I Trinchero R Piemonte Infective Endocarditis Study Group New trends in the epidemiological and clinical features of infective endocarditis: results of a multicenter prospective study Ital Heart J 2004 5 249 256 15185882

[B15] Scudeller L Badano L Crapis M Pagotto A Viale P Population-based surveillance of infectious endocarditis in an Italian region Arch Intern Med 2009 169 1720 1723 10.1001/archinternmed.2009.307 19822831

[B16] Fernández-Hidalgo N Almirante B Tornos P González-Alujas MT Planes AM Larrosa MN Sambola A Igual A Pahissa A Prognosis of left-sided infective endocarditis in patients transferred to a tertiary-care hospital-prospective analysis of referral bias and influence of inadequate antimicrobial treatment Clin Microbiol Infect 2010 in press 10.1111/j.1469-0691.2010.03314.x 20636419

[B17] Kanafani ZA Kanj SS Cabell CH Cecchi E de Oliveira Ramos A Lejko-Zupanc T Pappas PA Giamerellou H Gordon D Michelet C Muñoz P Pachirat O Peterson G Tan RS Tattevin P Thomas V Wang A Wiesbauer F Sexton DJ Revisiting the effect of referral bias on the clinical spectrum of infective endocarditis in adults Eur J Clin Microbiol Infect Dis 2010 29 1203 1210 10.1007/s10096-010-0983-2 20549531

[B18] Evangelista A Gonzalez-Alujas MT Echocardiography in infective endocarditis Heart 2004 90 614 617 10.1136/hrt.2003.029868 15145856 PMC1768290

[B19] Durack DT Lukes AS Bright DK New criteria for diagnosis of infective endocarditis: utilization of specific echocardiographic findings Am J Med 1994 96 200 209 10.1016/0002-9343(94)90143-0 8154507

[B20] Hasbun R Vikram HR Barakat LA Buenconsejo J Quagliarello VJ Complicated left-sided native valve endocarditis in adults. Risk classification for mortality JAMA 2003 289 1933 1940 10.1001/jama.289.15.1933 12697795

[B21] Durante-Mangoni E Bradley S Selton-Suty C Tripodi MF Barsic B Bouza E Cabell CH Ramos AI Fowler V Jr Hoen B Koneçny P Moreno A Murdoch D Pappas P Sexton DJ Spelman D Tattevin P Miró JM van der Meer JT Utili R International Collaboration on Endocarditis Prospective Cohort Study Group Current features of infective endocarditis in elderly patients: results of the International Collaboration on Endocarditis Prospective Cohort Study Arch Intern Med 2008 168 2095 2103 10.1001/archinte.168.19.2095 18955638

[B22] Di Salvo G Thuny F Rosenberg V Pergola V Belliard O Derumeaux G Cohen A Iarussi D Giorgi R Casalta JP Caso P Habib G Endocarditis in the elderly: clinical, echocardiographic, and prognostic features Eur Heart J 2003 24 1576 1583 10.1016/S0195-668X(03)00309-9 12927193

[B23] Choussat R Thomas D Isnard R Michel PL Iung B Hanania G Mathieu P David M du Roy de Chaumaray T De Gevigney G Le Breton H Logeais Y Pierre-Justin E de Riberolles C Morvan Y Bischoff N Perivalvular abscesses associated with endocarditis; clinical features and prognostic factors of overall survival in a series of 233 cases. Perivalvular Abscesses French Multicentre Study Eur Heart J 1999 20 232 241 10.1053/euhj.1998.1240 10082156

[B24] Sanfilippo AJ Picard MH Newell JB Rosas E Davidoff R Thomas JD Weyman AE Echocardiographic assessment of patients with infectious endocaditis: prediction of risk for complications J Am Coll Cardiol 1991 18 1191 1199 10.1016/0735-1097(91)90535-H 1918695

[B25] Akowuah EF Davies W Oliver S Stephens J Riaz I Zadik P Cooper G Prosthetic valve endocarditis: early and late outcome following medical or surgical treatment Heart 2003 89 269 272 10.1136/heart.89.3.269 12591827 PMC1767609

[B26] Tornos P Iung B Permanyer-Miralda G Baron G Delahaye F Gohlke-Bärwolf Ch Butchart EG Ravaud P Vahanian A Infective endocarditis in Europe: lessons from the Euro heart survey Heart 2005 91 571 575 10.1136/hrt.2003.032128 15831635 PMC1768869

[B27] Hoen B Alla F Selton-Suty C Béguinot I Bouvet A Briançon S Casalta JP Danchin N Delahaye F Etienne J Le Moing V Leport C Mainardi JL Ruimy R Vandenesch F Association pour l'Etude et la Prévention de l'Endocardite Infectieuse (AEPEI) Study Group Changing profile of infective endocarditis: results of a 1-year survey in France JAMA 2002 288 75 81 10.1001/jama.288.1.75 12090865

[B28] Chu VH Cabell CH Benjamin DK Jr Kuniholm EF Fowler VG Jr Engemann J Sexton DJ Corey GR Wang A Early predictors of in-hospital death in infective endocarditis Circulation 2004 109 1745 1749 10.1161/01.CIR.0000124719.61827.7F 15037538

